# Influence of sex on the association between body mass index and frequency of upper gastrointestinal symptoms

**DOI:** 10.1002/jgh3.12368

**Published:** 2020-06-05

**Authors:** Kyohei Ogisu, Atsuhiro Masuda, Tsuyoshi Fujita, Yukinao Yamazaki, Masao Kobayashi, Shuichi Terao, Tsuyoshi Sanuki, Akihiko Okada, Masayasu Adachi, Yoshifumi Arisaka, Haruka Miyazaki, Hayato Yoshinaka, Hiromu Kutsumi, Eiji Umegaki, Yuzo Kodama

**Affiliations:** ^1^ Division of Gastroenterology, Department of Internal Medicine Kobe University Graduate School of Medicine Kobe Japan; ^2^ Department of Gastroenterology Nissay Hospital Osaka Japan; ^3^ Department of Health Care Yodogawa Christian Hospital Osaka Japan; ^4^ Department of Gastroenterology Fukui Red Cross Hospital Fukui Japan; ^5^ Department of Health Care Kyoto Second Red Cross Hospital Kyoto Japan; ^6^ Department of Gastroenterology Kakogawa Central City Hospital Kakogawa Japan; ^7^ Department of Gastroenterology Kita‐Harima Medical Center Ono Japan; ^8^ Department of Gastroenterology Saiseikai Nakatsu Hospital Osaka Japan; ^9^ Department of Gastroenterology Hotel Okura Kobe Clinic Kobe Japan; ^10^ Clinical Research and Medical Innovation Center Shiga University Medical Science Otsu Japan

**Keywords:** body mass index, dyspeptic symptoms, sex difference, reflex symptoms, upper gastrointestinal symptoms

## Abstract

**Background and Aim:**

Upper gastrointestinal symptoms (UGSs), including reflux and dyspeptic symptoms (postprandial distress syndrome [PDS] and epigastric pain syndrome [EPS]), affect health‐related quality of life. However, the influence of sex on the relationship between body mass index (BMI) and UGSs remains controversial. This study investigates the influence of sex on this association in healthy subjects.

**Methods and Results:**

We utilized the database of a prospective, multicenter, cohort study of 7112 subjects who underwent upper endoscopy for health screening. A multivariable logistic regression analysis was conducted to assess the association between BMI and UGSs stratified by sex, adjusting for clinical features. The influence of sex on the association between the overlapping of UGSs and BMI in symptomatic subjects was also investigated. Reflux symptoms were significantly associated with high BMI (multivariable odds ratio [OR] 1.36; 95% confidence interval [CI] 1.10–1.67, *P* = 0.004). PDS symptoms were significantly associated with low BMI (OR 2.37; 95% CI 1.70–3.25; *P* < 0.0001), but EPS symptoms were not associated with BMI. The association between reflux symptoms and higher BMI was limited to men (men: OR 1.40; 95% CI 1.10–1.77; *P* = 0.005, women: *P* = 0.40). sex did not influence the association between the presence of PDS symptoms and lower BMI. The percentage of overlapping of all three symptoms (reflux, PDS, and EPS) was higher in women than in men (19.9% [58/292] *vs* 10.5% [49/468], *P* = 0.0002).

**Conclusions:**

The influence of BMI on the presence of UGSs was significantly different according to sex in this large‐scale cohort.

## Graphical abstract

This study aimed to investigate the influence of sex on the association between body mass index (BMI) and the presence of upper gastrointestinal symptoms using healthy check‐up subjects. The association between reflux symptoms and higher BMI was limited to males, although sex did not influence the association between the presence of functional dyspepsia (FD) symptoms and lower BMI. The percentage of overlapping of reflux and FD symptoms was higher in women than men.

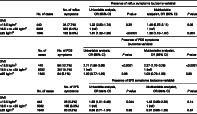

## Introduction

Upper gastrointestinal symptoms (UGSs), including abdominal pain or discomfort, heartburn, postprandial fullness, and early satiety, are often found in the general population, and the incidence of these symptoms is increasing.^1^, ^2^ If these symptoms become frequent or severe, patients may experience reduced health‐related quality of life (HRQOL).^3^, ^4^, ^5^ Heartburn is the major symptom of gastroesophageal reflux disease (GERD).^6^, ^7^ Other symptoms such as dyspeptic symptoms (postprandial fullness and early satiety) and epigastric pains are common in functional dyspepsia (FD).^8^ These symptoms are not always independent and sometimes overlap and influence each other.^5^, ^9^, ^10^, ^11^ This overlapping makes not only the diagnosis complex but also the choice of appropriate treatment more challenging.

Increased body mass index (BMI) is a well‐known risk factor for GERD symptoms and erosive esophagitis (EE).^12^, ^13^ Generally, proton‐pump inhibitors (PPIs) are effective for treating GERD symptoms.^6^, ^14^, ^15^ A previous report showed that PPIs were effective for GERD with low and high BMI,^16^ but whether low BMI is a risk factor for GERD symptoms remains unknown. Moreover, reports on the association between FD symptoms and BMI are limited. One survey in seven western countries and Japan reported that higher BMI was related to higher frequency of UGSs.^17^ On the contrary, Pluart *et al*. reported that low and high BMIs are related to higher frequency of FD symptoms in women.^18^ To date, there is no consistent evidence on the relationship between UGSs and BMI.

Evidence of the association between sex and frequency of UGSs is still scarce. One report showed that EE is more common in young women^19^; conversely, another report showed that, although similar proportions of men and women have GERD symptoms, an increasingly greater proportion of men have EE.^20^ A western population‐based study showed that women were more at risk of FD.^21^ However, other reports have not shown sex differences in FD prevalence.^22^ Although sex may influence the association between BMI and UGSs, the evidence is not consistent. Therefore, we aimed to clarify the influence of sex on the association between BMI and UGSs using data from thousands of individuals who underwent health screening in a multicenter prospective study.

## Materials

### *Subjects*

We utilized the database of a prospective, multicenter, cohort study (Upper Gastrointestinal Disease [UGID] study) of 8889 subjects enrolled during 2013–2014, who underwent upper endoscopy for health screening. Of the 8889 subjects, 7112 were eligible. We excluded 597 individuals taking PPI or H2 receptor antagonist (H2RA), 165 taking other digestive agents (prokinetic and mucosal protective agents, etc.), 811 with incomplete questionnaire data, 59 with incomplete endoscopic findings, 66 who had undergone gastrectomy, 37 with gastric ulcer, 31 with duodenal ulcer, 9 with gastric cancer, and 1 with esophageal cancer. One participant was excluded after withdrawing consent.

### *Prospective questionnaire and endoscopic findings*

Data were collected using a prospective questionnaire (Data S1), including age, height, bodyweight, sex, frequency of UGSs (reflux, postprandial distress syndrome [PDS], and epigastric pain syndrome [EPS] symptoms), current smoking status, and current average daily alcohol consumption (0, <20, or ≥20 g/day; cut‐off value of 20 g/day is determined according to previous Japanese epidemiological studies).^23^, ^24^ Hiatal hernia (HH) was defined as the proximal dislocation of gastroesophageal junction (GEJ) >2 cm above diaphragmatic indentation. The severity of HH was categorized by the length of the proximal dislocation of GEJ, with 2–4 cm considered mild and >4 cm severe.

According to the Los Angeles classification of the severity of reflux esophagitis,^25^ EE was divided endoscopically into the following four grades: A (mild) to D (severe). Atrophic gastritis was endoscopically diagnosed, and the endoscopic extent of atrophic mucosa was graded according to Kimura‐Takemoto classification from C‐1 to O‐3.^26^ Subjects with atrophic mucosa grades C‐2, C‐3, O‐1, O‐2, and O‐3 were described as positive for atrophic gastritis. We have evaluated trait anxiety using the State‐Trait Anxiety Inventory (STAI) questionnaire.^27^ A high STAI score was defined as ≥44 in men and ≥45 in women.

This study was conducted in accordance with the Declaration of Helsinki and its amendments (UMIN‐CTR ID: 000022504). The study protocol was approved by the Ethics Committee of each institution (Yodogawa Christian Hospital, Fukui Red Cross Hospital, Kyoto Second Red Cross Hospital, Kakogawa Central City Hospital, Kita‐Harima Medical Center, Saiseikai Nakatsu Hospital, and Hotel Okura Kobe Clinic and Kobe University; No. 170223). Written informed consent was obtained from all participants.

### *Definition of* *UGSs*

In a prospective questionnaire, the frequency of UGSs in the past 3 months was investigated (i.e., never, <1 day/month, 1 day/month, 2–3 days/month, 1 day/week, >1 day/week, or every day) according to our previous report.^28^ Reflux symptoms were measured according to the frequency of heartburn and/or acid regurgitation. PDS symptoms were measured according to the frequency of bothersome postprandial fullness and/or early satiation. EPS symptoms were measured according to the frequency of epigastric pain and/or epigastric burning.

### *Statistical analyses*

All statistical analyses were conducted using JMP version 11 (SAS Institute, Cary, NC, USA), and all *P* values were two‐sided. Our primary hypothesis testing used the likelihood test in a binary logistic regression model to assess the association between UGSs and BMI. Binary categorical variables ([presence and absence] of UGSs) were used as outcome variables. The multivariable model initially included age group; sex; current smoking status; alcohol consumption ≥20 g/day; and presence of EE, atrophic gastritis, HH, and high STAI score. A backward stepwise elimination with a threshold of *P* = 0.05 was used to select variables in the final models. Given that we tested three primary hypotheses (reflux, PDS, and EPS symptoms as outcome variables), we corrected the statistical significance level to *P* = 0.017 (= 0.05/3). Then, we constructed a binary logistic regression model to assess the association between UGSs and BMI, stratified by sex. For secondary analyses, we recognized multiple comparisons inherent in the subgroup analyses and interpreted our data cautiously. To assess associations between categorical data, the chi‐square test (or Fisher's exact test, as appropriate) was performed. To compare mean age, mean STAI score, and frequency scale for the symptoms of GERD (FSSG) score, *t*‐test or analysis of variance, assuming equal variances, was carried out.

## Results

### *Clinical features of subjects according to* *BMI* *in the* *UGID* *study*

In the analysis of the clinical features of the 7112 subjects stratified by BMI (Table 1), the proportion of participants in each BMI category was as follows: BMI <18.5, 440 (6.2%); BMI ≥18.5 to <25, 5032 (70.7%); and BMI ≥25, 1640 (23.1%). Age, age group, sex, smoking status, alcohol consumption, EE, HH, and FSSG scores were associated with BMI. Youthfulness and women were associated with low BMI (*P* < 0.0001 and *P* < 0.0001, respectively). Current smoking and high amount of alcohol consumption were associated with high BMI (*P* < 0.0001). STAI score was significantly associated only in women (women: *P* = 0.012, men: *P* = 0.63). Although participants with low BMI had higher STAI scores (continuous variable), high STAI scores were not significantly associated with BMI (*P* = 0.38). Moreover, sex showed no influence on the relationship between BMI and high STAI score (men: *P* = 0.86, women: *P* = 0.15). A high FSSG score was associated with a low BMI (*P* = 0.001).

**Table 1:** Clinical features of participants according to body mass index in the Upper Gastrointestinal Disease study

		Body mass index			
	Total no.	<18.5 kg/m^2^	18.5–25 kg/m^2^	>25 kg/m^2^	*P* value
All cases	7112	440 (6.2%)	5032 (70.7%)	1640 (23.1%)	
Age (mean ± SD)	52.1 ± 9.9	49.6 ± 10.0	52.2 ± 10.0	52.5 ± 9.5	<0.0001
Age group					<0.0001
≤39 years	693 (9.7%)	71 (16.1%)	497 (9.9%)	125 (7.6%)	
40–59 years	4760 (66.9%)	296 (67.3%)	3314 (65.9%)	1150 (70.1%)	
≥60 years	1659 (23.3%)	73 (16.6%)	1221 (24.3%)	365 (22.3%)	
Sex					<0.0001
Male	4473 (62.9%)	89 (20.2%)	3073 (61.1%)	1311 (79.9%)	
Female	2639 (37.1%)	351 (79.8%)	1959 (38.9%)	329 (20.1%)	
Smoking status					<0.0001
Never	3458 (48.6%)	323 (73.4%)	2489 (49.5%)	646 (39.4%)	
Former	2494 (35.1%)	71 (16.1%)	1735 (34.4%)	688 (42.0%)	
Current	1160 (16.3%)	46 (10.5%)	808 (16.1%)	306 (18.6%)	
Alcohol consumption (≥20 g/day)					<0.0001
Presence	1909 (26.8%)	49 (11.1%)	1318 (26.2%)	542 (33.0%)	
Absence	5203 (73.2%)	391 (88.9%)	3714 (73.8%)	1098 (67.0%)	
STAI score (mean ± SD)	41.5 ± 9.9	43.3 ± 10.4	41.5 ± 9.8	41.1 ± 9.9	0.38
High STAI score					0.15
Presence	2693 (37.9%)	184 (41.8%)	1907 (37.9%)	602 (36.7%)	
Absence	4419 (62.1%)	256 (58.2%)	3125 (62.1%)	1038 (63.3%)	
Erosive esophagitis					<0.0001
Presence	1201 (16.9%)	23 (5.2%)	731 (14.5%)	447 (27.3%)	
Absence	5911 (83.1%)	417 (94.8%)	4301 (85.5%)	1193 (72.7%)	
Atrophic gastritis					0.08
Presence	2715 (38.2%)	153 (34.8%)	1904 (37.8%)	658 (40.1%)	
Absence	4397 (61.8%)	287 (65.2%)	3128 (62.2%)	982 (59.9%)	
Hiatal hernia					<0.0001
Presence	2701 (29.1%)	67 (15.2%)	1388 (27.6%)	616 (37.6%)	
Absence	5041 (70.9%)	373 (84.8%)	3644 (72.4%)	1024 (62.4%)	
FSSG total score (mean ± SD)	5.19 ± 5.42	6.25 ± 6.57	5.09 ± 5.31	5.24 ± 5.37	0.0001

% indicates the proportion of cases with a special clinical feature in participants according to body mass index. FSSG, frequency scale for the symptoms of gastroesophageal reflux disease; STAI, State‐Trait Anxiety Inventory.

### *Relationship between* *BMI* *and frequency of* *UGSs*

In the analysis of the frequency of UGSs stratified by BMI (Table 2), the frequency of reflux and PDS symptoms was significantly associated with BMI (*P* < 0.0001), whereas that of EPS symptoms was not (*P* = 0.19). Reflux symptoms were more frequently observed in high‐BMI subjects, whereas PDS and EPS symptoms were more frequently observed in low‐BMI subjects. The frequency of dyspeptic symptoms (PDS or EPS symptoms) and UGSs was also significantly associated with BMI (*P* < 0.0001, *P* = 0.0004, respectively).

**Table 2:** Relationship between body mass index and frequency of upper gastrointestinal symptoms

		Body mass index			
	Total no.	<18.5 kg/m^2^	18.5–25 kg/m^2^	>25 kg/m^2^	*P* value
Frequency of reflux symptoms					<0.0001
None	4456 (62.7%)	292 (66.4%)	3224 (64.1%)	940 (57.3%)	
<1 day/week	2137 (30.0%)	114 (25.9%)	1486 (29.5%)	537 (32.7%)	
≥1 day/week	519 (7.3%)	34 (7.7%)	322 (6.4%)	163 (9.9%)	
Frequency of PDS symptoms					<0.0001
None	5097 (71.7%)	269 (61.1%)	3613 (71.8%)	1215 (74.1%)	
<1 day/week	1618 (22.8%)	115 (26.1%)	1162 (23.1%)	341 (20.8%)	
≥1 day/week	397 (5.6%)	56 (12.7%)	257 (5.1%)	84 (5.1%)	
Frequency of EPS symptoms					0.19
None	5606 (78.9%)	334 (75.9%)	3965 (78.8%)	1307 (79.7%)	
<1 day/week	1265 (17.8%)	83 (18.9%)	902 (17.9%)	280 (17.1%)	
≥1 day/week	241 (3.4%)	23 (5.2%)	165 (3.3%)	53 (3.2%)	
Frequency of dyspeptic symptoms					<0.0001
None	4488 (63.1%)	238 (54.1%)	3183 (63.3%)	1067 (65.1%)	
<1 day/week	2111 (29.7%)	139 (31.4%)	1508 (30.0%)	464 (28.3%)	
≥1 day/week	513 (7.2%)	63 (14.3%)	341 (6.8%)	109 (6.7%)	
Frequency of upper gastrointestinal symptoms					0.0004
None	3639 (51.2%)	210 (47.8%)	2625 (52.2%)	804 (49.0%)	
<1 day/week	2713 (38.1%)	161 (36.6%)	1912 (38.0%)	640 (39.0%)	
≥1 day/week	760 (10.7%)	69 (15.7%)	495 (9.8%)	196 (12.0%)	

EPS, epigastric pain syndrome; PDS, postprandial distress syndrome.

### *Logistic regression analysis of the relationship between* *BMI* *and frequency of* *UGSs*

The results of the analysis of the relationship between BMI and UGSs of >1 day/week are shown in Table 3. The presence of reflux symptoms (>1 day/week) was significantly associated with high BMI (multivariable odds ratio [OR] 1.36; 95% confidence interval [CI] 1.10–1.67, *P* = 0.004) and showed a marginally significant association with low BMI (OR 1.46; 95% CI 0.97–2.11; *P* = 0.06, not significant in the univariate analysis). The presence of PDS symptoms (>1 day/week) was significantly associated with lower BMI (OR 2.37; 95% CI 1.70–3.25; *P* < 0.0001). The presence of EPS symptoms (>1 day/week) was not associated with BMI (*P* > 0.05).

**Table 3:** Logistic regression analysis for assessing the relationship between body mass index and upper gastrointestinal symptoms with frequency of >1 day/week

			Presence of reflux symptoms (outcome variable)			
	No. of cases	No. of reflux symptoms	Univariable analysis, OR (95% CI)	*P* value	Multivariable analysis†, OR (95% CI)	*P* value
BMI						
<18.5 kg/m^2^	440	34 (7.7%)	1.22 (0.83–1.74)	0.29	1.46 (0.97–2.11)	0.06
18.5 ≤ to <25 kg/m^2^	5032	322 (6.4%)	1 (ref)		1 (ref)	
≥25 kg/m^2^	1640	163 (9.9%)	1.61 (1.32–1.96)	<0.0001	1.36 (1.10–1.67)	0.004

			Presence of PDS symptoms (outcome variable)			
	No. of cases	No. of PDS symptoms	Univariable analysis, OR (95% CI)	*P* value	Multivariable analysis†, OR (95% CI)	*P* value
BMI						
<18.5 kg/m^2^	440	56 (12.7%)	2.71 (1.98–3.66)	<0.0001	2.37 (1.70–3.25)	<0.0001
18.5 ≤ to <25 kg/m^2^	5032	257 (5.1%)	1 (ref)		1 (ref)	
≥25 kg/m^2^	1640	84 (5.1%)	1.00 (0.77–1.29)	0.98	1.03 (0.79–1.33)	0.83

			Presence of EPS symptoms (outcome variable)			
	No. of cases	No. of EPS symptoms	Univariable analysis, OR (95% CI)	*P* value	Multivariable analysis†, OR (95% CI)	*P* value
BMI						
<18.5 kg/m^2^	440	23 (5.2%)	1.63 (1.01–2.49)	0.044	1.43 (0.88–2.23)	0.14
18.5 ≤ to <25 kg/m^2^	5032	165 (3.3%)	1 (ref)		1 (ref)	
≥25 kg/m^2^	1640	53 (3.2%)	0.99 (0.71–1.34)	0.93	0.91 (0.65–1.25)	0.57

† The OR initially included age, sex, presence of current smoking, presence of alcohol consumption ≥20 g/day, presence of erosive esophagitis, presence of atrophic gastritis, presence of hiatal hernia, and presence of a high State‐Trait Anxiety Inventory score. A backward stepwise elimination with a threshold of *P* = 0.05 was used to select variables in the final models. Given that we tested three primary hypotheses (for reflux, PDS, and EPS symptoms as outcome variables), we corrected the statistical significance level to *P* = 0.017 (= 0.05/3) by simple Bonferroni correction. BMI, body mass index; CI, confidence interval; EPS, epigastric pain syndrome; OR, odds ratio; PDS, postprandial distress syndrome.

### *Logistic regression analysis of the relationship between* *BMI* *and frequency of* *UGSs* *stratified by sex*

The results of the analysis of the influence of sex on the relationship between BMI and frequency of UGSs are presented in Table 4. The presence of reflux symptoms (>1 day/week) was significantly associated with high BMI in men (OR 1.40; 95% CI 1.10–1.77; *P* = 0.005) but not in women (OR 1.22; 95% CI 0.76–1.90; *P* = 0.40).

**Table 4:** Logistic regression analysis to assess the relationship between body mass index and upper gastrointestinal symptoms with frequency of >1 day/week stratified by sex

Men			Women		
	% of cases	Multivariable analysis†, OR (95% CI)	*P* value	% of cases	Multivariable analysis†, OR (95% CI)	*P* value
BMI	Presence of reflux symptoms (outcome variable)					
<18.5 kg/m^2^	9.0% (8/89)	1.79 (0.78–3.58)	0.16	7.4% (26/351)	1.34 (0.84–2.06)	0.22
18.5 ≤ to <25 kg/m^2^	6.7% (207/3073)	1 (ref)		5.9% (115/1959)	1 (ref)	
≥25 kg/m^2^	10.5% (137/1311)	1.40 (1.10–1.77)	0.005	7.9% (26/329)	1.22 (0.76–1.90)	0.40
BMI	Presence of PDS symptoms (outcome variable)					
<18.5 kg/m^2^	13.5% (12/89)	4.36 (2.18–8.04)	0.0001	12.5% (44/351)	1.94 (1.32–2.79)	0.0009
18.5 ≤ to <25 kg/m^2^	4.0% (123/3073)	1 (ref)		6.8% (134/1959)	1 (ref)	
≥25 kg/m^2^	5.2% (68/1311)	1.22 (0.89–1.65)	0.22	4.9% (16/329)	0.69 (0.39–1.15)	0.16
BMI	Presence of EPS symptoms (outcome variable)					
<18.5 kg/m^2^	2.3% (2/89)	1.02 (0.17–3.38)	0.97	6.0% (21/351)	1.46 (0.86–2.36)	0.15
18.5 ≤ to <25 kg/m^2^	2.8% (85/3073)	1 (ref)		4.1% (80/1996)	1 (ref)	
≥25 kg/m^2^	3.0% (39/1311)	0.88 (0.59–1.29)	0.52	4.3% (14/340)	1.00 (0.53–1.75)	0.99

† The OR initially included age group, presence of current smoking, presence of alcohol consumption ≥20 g/day, presence of erosive esophagitis, presence of atrophic gastritis, presence of hiatal hernia, and presence of high State‐Trait Anxiety Inventory score. A backward stepwise elimination with a threshold of *P* = 0.05 was used to select variables in the final models. Given that we tested three primary hypotheses (for reflux, PDS, and EPS symptoms as outcome variables), we corrected the statistical significance level to *P* = 0.017 (= 0.05/3) by simple Bonferroni correction. BMI, body mass index; CI, confidence interval; EPS, epigastric pain syndrome; OR, odds ratio; PDS, postprandial distress syndrome.

Regardless of sex, the presence of PDS symptoms (>1 day/week) was significantly associated with low BMI (men: OR 4.36; 95% CI 2.18–8.04; *P* = 0.0001, women: OR 1.94; 95% CI 1.32–2.79; *P* = 0.0009).

### *Relationship between the overlapping of* *UGSs* *and* *BMI*

**Figure 1:**
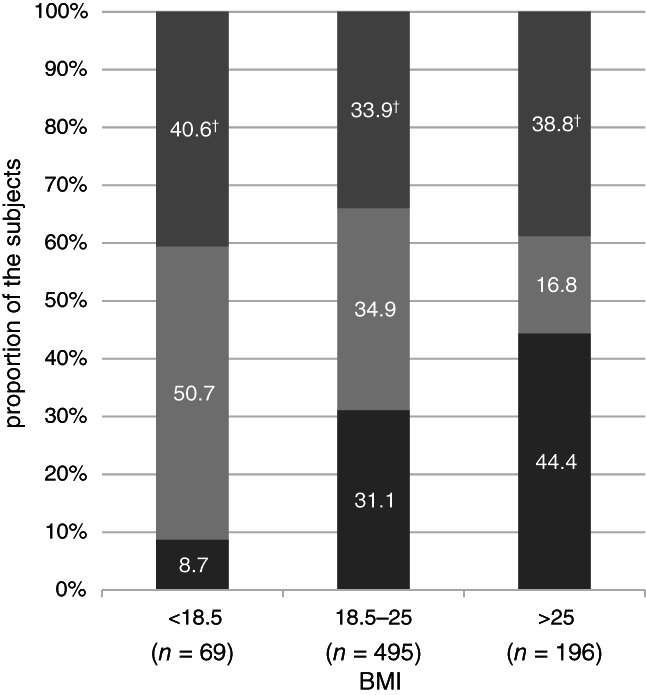
Relationship between overlapping of upper gastrointestinal symptoms (UGSs) and body mass index (BMI) in subjects having UGSs with frequency of >1 day/week. Reflux only: reflux symptoms without dyspeptic symptoms; 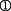. Functional dyspepsia (FD) only: dyspeptic symptoms without reflux symptoms; 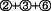. Overlap: overlap of reflux and dyspeptic symptoms; 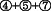. ( ), Overlap; ( ), FD only; (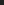 ), reflux only. †*P* < 0.01.

The relationship between the overlapping of UGSs and BMI was analyzed using data from subjects with at least one UGS; subjects without symptoms were excluded. A significant association was found between BMI and the overlapping of the UGSs (*P* = 0.001, Fig. 1, Table S1, Supporting information). Higher BMI was associated with higher proportions of reflux symptoms without dyspeptic symptoms (OR 1.40; 95% CI 1.06–1.85; *P* = 0.02). On the contrary, lower BMI was associated with higher proportions of dyspeptic symptoms without reflux symptoms (OR 1.94; 95% CI 1.29–2.86; *P* = 0.002, Table S3).

### *Influence of sex on the relationship between the overlapping of* *UGSs* *and* *BMI*

**Figure 2:**
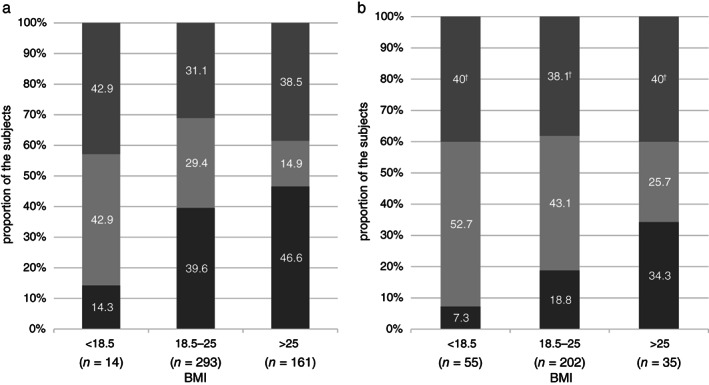
(a) Relationship between overlapping of upper gastrointestinal symptoms (UGSs) and body mass index (BMI) in men having UGSs with frequency of >1 day/week. Reflux only: reflux symptoms without dyspeptic symptoms; 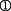. Functional dyspepsia (FD) only: dyspeptic symptoms without reflux symptoms; 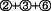. Overlap: overlap of reflux and dyspeptic symptoms; 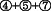. Overlap: overlap of reflux and dyspeptic symptoms; 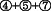. (b) Relationship between overlapping of UGSs and BMI in women having UGSs with frequency of >1 day/week. Reflux only: reflux symptoms without dyspeptic symptoms; 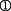. FD only: dyspeptic symptoms without reflux symptoms; 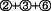. Overlap: overlap of reflux and dyspeptic symptoms; 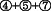. Overlap: overlap of reflux and dyspeptic symptoms; 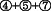. (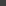 ), Overlap; (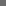 ), FD only; (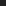), reflux only.

There was a significant association between BMI and the overlapping of UGSs according to sex (men: *P* = 0.14 *vs* women *P* = 0.004, Fig. 2).

Regardless of sex, higher BMI was associated with higher proportions of reflux symptoms without dyspeptic symptoms, whereas lower BMI was associated with higher proportions of dyspeptic symptoms without reflux symptoms. The association of high BMI and reflux symptoms was slightly stronger in men than in women. In contrast, the association between low BMI and dyspeptic symptoms was slightly stronger in women than in men.

Details of the association between BMI and overlapping of UGSs are shown in Table S1. The percentage of overlapping of all three symptoms (reflux, PDS, and EPS) was the highest in low‐BMI subjects (low BMI, 20.3% [14/69] *vs* normal BMI, 13.9% [69/495] *vs* high BMI, 12.2% [24/196]; *P* = 0.01) and was higher in women than in men (19.9% [58/292] *vs* 10.5% [49/468], *P* = 0.0002).

## Discussion

The major findings of this study are as follows: First, reflux symptoms were significantly associated with high BMI, whereas PDS symptoms were significantly associated with low BMI. However, EPS symptoms were not associated with BMI. Second, the association between reflux symptoms and high BMI was limited to men. However, sex did not influence the association between PDS symptoms and lower BMI. Third, a significant association was found between BMI and the overlapping of UGSs. Among symptomatic subjects, higher BMI was associated with higher proportions of reflux symptoms without FD symptoms (>1 day/week), whereas lower BMI was associated with higher proportions of dyspeptic symptoms without reflux symptoms (>1 day/week). The percentage of overlapping of all three symptoms was higher in low‐BMI subjects than in normal‐ and high‐BMI subjects. In addition, the percentage of overlapping of all three symptoms was higher in women than in men. The influence of BMI on the presence of UGSs was significantly different between sexs.

There is limited evidence regarding the relationship between the prevalence of reflux symptoms and BMI.^29^ However, there is consistent evidence in patients with erosive GERD and endoscopically confirmed mucosal breaks on the GEJ. In erosive GERD, higher BMI was significantly associated with its prevalence than normal or lower BMI.^30^, ^31^, ^32^ In contrast, other reports have shown that low BMI is associated with non‐erosive GERD than with erosive GERD.^33^ Moreover, according to a survey of Korean nurses, which was limited to women subjects, both high and low BMIs were associated with reflux symptoms.^34^ Thus, the presence of erosion on the GEJ or sex might influence the association between BMI and prevalence of reflux symptoms. In our study, reflux symptoms were associated with high BMI only in men and not in women (Table 4). In the analysis of patients with EE, reflux symptoms were associated with high BMI. In our study, the reflux symptoms without EE had a tendency toward low BMI (Table S2).

There is not much evidence regarding the relationship between prevalence of FD and BMI. To the best of our knowledge, there are only a few reports on its association. In a report from Iran, BMI and prevalence of FD were not related.^35^ In a Western population‐based study, women were more likely to develop FD and less likely to be cured than men.^21^ Early satiety, a symptom of PDS, has been associated with low BMI and weight loss.^36^, ^37^, ^38^, ^39^ Pluart *et al*. reported that both low and high BMIs are associated with FD in women.^18^ In our study, low BMI was a significant risk factor for PDS symptoms but not for EPS symptoms. Sex differences were not identified in its association.

Generally, the proportion of individuals with a BMI of >30 is small within a Japanese population. Indeed, only several subjects had BMI >30 in this study. Although the mechanisms underlying the association between FD symptoms and BMI are not fully understood, the association with weight loss may be related to gastric adaptive relaxation and hypersensitivity in FD.^38^ A study on FD has suggested that, although early satiety and impaired fundic accommodation in some subjects would lead to undernutrition and therefore an underweight status, those without impairment would, in fact, present with a normal weight or be overweight.^18^

Overlapping of reflux and dyspeptic symptoms were often observed and may lead to a decrease in HRQOL. The Rome III committee concluded that patients with a chief complaint of typical heartburn almost certainly had GERD and should be distinguished from dyspeptic patients.^10^ However, several studies have shown that reflux symptoms coexist in a significant proportion of dyspeptic patients.^40^, ^41^ In one Japanese cohort, 24.6% of patients with UGSs had both GERD and FD symptoms.^5^ Another report from Netherlands showed that 25% of 263 GERD patients had FD symptoms.^42^ These reports suggested that GERD patients might have overlapping FD symptoms with some frequencies. In our study, 35.8% (272/760) of subjects with UGSs had an overlap of GERD and FD symptoms (Table S1). We speculated that the prevalence of these overlapping symptoms was higher in our study than in previous reports because our study was a symptom‐based study involving individuals who underwent health screening and did not strictly meet the FD and GERD criteria.

To the best of our knowledge, there are no reports about the influence of BMI on overlapping UGSs. In our study, as BMI increased, the ratio of reflux symptoms without FD symptoms (>1 day/week) increased, whereas that of FD symptoms without reflux symptoms (>1 day/week) decreased (Fig. 1 and Table S3). Reflux symptoms without FD symptoms (>1 day/week) were associated with high BMI only in men. FD symptoms without reflux symptoms (>1 day/week) were significantly more frequent in low‐BMI subjects than in normal‐ or high‐BMI subjects, and its relationship was observed only in women (Table S4).

The large amount of data from a prospective multicenter cohort study strengthens these findings. The size and comprehensiveness of this database enabled us to elucidate the influence of BMI and sex on the frequency of UGSs. All the subjects underwent upper endoscopy. Given that this study utilized all the registered data in the database during the study period, we did not perform any sample size calculation at the planning stage. In a post hoc power analyses, the statistical power of >95% at alpha = 5% was revealed for the actual observed proportions of 9.9% (163/1640) and 6.4% (322/5032), as shown in Table 3, and of 10.5% (137/1311) and 6.7% (207/3073), as shown in Table 4. These demonstrated that the present study was sufficiently powered to detect effect, at least for major findings.

Our study had several limitations. First, this was a cross‐sectional study, and the causal relationship between BMI and UGSs remains unknown. The UGID study is a 5‐year follow‐up study. In the future, we are planning to investigate the effect of BMI changes on the incidence of UGSs. Second, this was a symptom‐based study involving individuals who underwent health screening, and not all subjects met the diagnostic criteria of GERD or FD. Third, all subjects were Japanese, and only a few had BMI > 30. Differences in the relationship between prevalence of FD and BMI possibly exist among races, but we could not assess them. Third, the subjects of our study were not “true patients.” We focused on the frequency of UGSs in the general population influenced by BMI and sex, and the symptoms of our subjects were relatively mild. However, our study had a 5‐year‐follow‐up duration, and at 2 years after the start of this study (follow‐up number of subjects without any types of digestive agents at the first year: *n* = 4019), 154 subjects started regular medications of PPI or H2RA. This result suggested that, although most of our subjects had mild symptoms, some of the “true patients” were included or were newly diagnosed during the follow‐up period. Furthermore, we did not confirm the *Helicobacter pylori* infection status of all subjects. Generally, it is known that *H. pylori* infection is associated with a decreased prevalence of GERD, which also affects FD symptoms. We have determined the *H. pylori* status through a questionnaire about abdominal symptoms (question item 6 in Data S1). A total of 1673 subjects were diagnosed with *H. pylori* infection by urea breath test (UBT) in other hospitals, whereas 1130 were negative. Other patients were not examined, and their status was unknown. In the analysis using our limited data, *H. pylori* infection status was not associated with each UGS and BMI by univariable analysis (data not shown).

In conclusion, the influence of BMI and the presence of UGSs significantly differ between sexs in this large population of individuals who underwent health screening. In addition, overlapping of UGSs is frequently seen, and the influence of BMI on the overlapping of UGSs also differed according to sex. The influence of BMI on the presence of UGSs is significantly different according to sex in this large‐scale cohort.

## Supporting information


**Table S1.** Details of overlap of upper abdominal symptoms stratified by body mass index in the subjects having upper abdominal symptoms of the frequency more than 1 day /week.
**Table S2.** Relationship between body mass index and the frequencies of reflux symptoms more than once a week according to the presence or absence of erosive esophagitis.
**Table S3.** Relationship of body mass index with the presence of reflux symptoms without FD symptoms or the presence of FD symptoms without reflux symptoms.
**Table S4.** Relationship of body mass index with the presence of reflux symptoms without FD symptoms or the presence of FD symptoms without reflux symptoms stratified by sex.

**Data S1.** Upper gastrointestinal disease study questionnaire.
