# Supplementary material for: Influence of sex on the association between body mass index and frequency of upper gastrointestinal symptoms
Source: JGH Open. 2020 Jun 5;4(5):937–44. doi: 10.1002/jgh3.12368 (PMC7578286; doi:10.1002/jgh3.12368)
Supplement: Supplementary file 1 — Table S1. Details of overlap of upper abdominal symptoms stratified by body mass index in the subjects having upper abdominal symptoms of the frequency more than 1 day /week. Table S2. Relationship between body mass index and the frequencies of reflux symptoms more than once a week according to the presence or absence of erosive esophagitis. Table S3. Relationship of body mass index with the presence of reflux symptoms without FD symptoms or the presence of FD symptoms without reflux symptoms. Table S4. Relationship of body mass index with the presence of reflux symptoms without FD symptoms or the presence of FD symptoms without reflux symptoms stratified by sex. [file JGH3-4-937-s001.zip › JGH3_12368_revision supplemental table_1-4_supplymentaly table_clear.docx]

Supplemental Table 1. Details of overlap of upper abdominal symptoms stratified by body mass index in the subjects having upper abdominal symptoms of the frequency more than 1 day /week

|  |  | Body Mass Index | | |
| --- | --- | --- | --- | --- |
|  | Total no. | <18.5 kg/m^2^ | 18.5-25 kg/m^2^ | >25 kg/m^2^ |
| **All subjects** | 760 | 69 | 495 | 196 |
| ①Reflux symptoms only | 247 (32.5%) | 6 (8.7%) | 154 (31.1%) | 87 (44.4%) |
| ②PDS symptoms only | 173 (22.8%) | 30 (43.5%) | 119 (24.0%) | 24 (12.2%) |
| ③EPS symptoms only | 50 (6.6%) | 3 (4.3%) | 42 (8.5%) | 5 (2.6%) |
| ④Reflux + PDS symptoms | 99 (13.0%) | 10 (14.5%) | 57 (11.5%) | 32 (16.3%) |
| ⑤Reflux + EPS symptoms | 66 (8.6%) | 4 (5.8%) | 42 (8.5%) | 20 (10.2%) |
| ⑥PDS + EPS symptoms | 18 (2.4%) | 2 (2.9%) | 12 (2.4%) | 4 (2.0%) |
| ⑦Reflux + PDS + EPS symptoms | 107 (14.1%) | 14 (20.3%) | 69 (13.9%) | 24 (12.2%) |
|  |  |  |  |  |
| **Men** | 468 | 14 | 293 | 161 |
| ①Reflux symptoms only | 193 (41.2%) | 2 (14.3%) | 116 (39.6%) | 75 (46.6%) |
| ②PDS symptoms only | 85 (18.2%) | 6 (42.9%) | 60 (20.5%) | 19 (11.8%) |
| ③EPS symptoms only | 26 (5.6%) | 0 (0.0%) | 24 (8.2%) | 2 (1.2%) |
| ④Reflux + PDS symptoms | 64 (13.7%) | 4 (28.6%) | 32 (10.9%) | 28 (17.4%) |
| ⑤Reflux + EPS symptoms | 46 (9.8%) | 0 (0.0%) | 30 (10.2%) | 16 (9.9%) |
| ⑥PDS + EPS symptoms | 5 (1.1%) | 0 (0.0%) | 2 (0.7%) | 3 (1.9%) |
| ⑦Reflux + PDS + EPS symptoms | 49 (10.5%) | 2 (14.3%) | 29 (9.9%) | 18 (11.2%) |
|  |  |  |  |  |
| **Women** | 292 | 55 | 202 | 35 |
| ①Reflux symptoms only | 54 (18.5%) | 4 (7.3%) | 38 (18.8%) | 12 (34.3%) |
| ②PDS symptoms only | 88 (30.1%) | 24 (43.6%) | 59 (29.2%) | 5 (14.3%) |
| ③EPS symptoms only | 24 (8.2%) | 3 (5.5%) | 18 (8.9%) | 3 (8.6%) |
| ④Reflux + PDS symptoms | 35 (12.0%) | 6 (10.9%) | 25 (12.4%) | 4 (11.4%) |
| ⑤Reflux + EPS symptoms | 20 (6.8%) | 4 (7.3%) | 12 (5.9%) | 4 (11.4%) |
| ⑥PDS + EPS symptoms | 13 (4.5%) | 2 (3.6%) | 10 (5.0%) | 1 (2.9%) |
| ⑦Reflux + PDS + EPS symptoms | 58 (19.9%) | 12 (21.8%) | 40 (19.8%) | 6 (17.1%) |

BMI, body mass index; EPS, epigastric pain syndrome; FD, functional dyspepsia; PDS, postprandial distress syndrome

Supplemental Table 2. Relationship between body mass index and the frequencies of reflux symptoms more than once a week according to the presence or absence of erosive esophagitis.

|  |  |  | Presence of reflux symptoms with erosive esophagitis | | | |
| --- | --- | --- | --- | --- | --- | --- |
|  | No. of | No. of | Univariable analysis  OR (95% CI) | *P* value | Multivariable analysis*  OR (95% CI) | *P* value |
|  | cases | Reflux  symptoms |  |  |  |  |
| BMI |  |  |  |  |  |  |
| <18.5 kg/m^2^ | 23 | 2 (8.7%) | 0.23 (0.04-0.72) | *P*=0.008 | 0.42 (0.07-1.36) | *P=0.17* |
| 18.5≤ to <25 kg/m^2^ | 731 | 99(13.5%) | 1 (ref) |  | 1 (ref) |  |
| ≥25 kg/m^2^ | 447 | 77 (17.2%) | 2.45 (1.80-3.32) | *P*<0.0001 | 1.96 (1.43-2.67) | *P<0.0001* |
|  |  |  | Presence of reflux symptoms without erosive esophagitis | | | |
|  | No. of | No. of | Univariable analysis  OR (95% CI) | *P* value | Multivariable analysis*  OR (95% CI) | *P* value |
|  | cases | Reflux  symptoms |  |  |  |  |
| BMI |  |  |  |  |  |  |
| <18.5 kg/m^2^ | 417 | 32 (7.7%) | 1.69 (1.13-2.45) | *P*=0.011 | 1.59 (1.05-2.34) | *P=0.03* |
| 18.5≤ to <25 kg/m^2^ | 4301 | 223 (5.2%) | 1 (ref) |  | 1 (ref) |  |
| ≥25 kg/m^2^ | 1193 | 86 (7.2%) | 1.19 (0.92-1.53) | *P*=0.18 | 1.22 (0.94-1.58) | *P*=0.14 |

*The odds ratio was initially included age group, sex, presence of current smoking, presence of alcohol consumption ≥20g/day, presence of atrophic gastritis, presence of hiatal hernia, and presence of high STAI score. A backward stepwise elimination with a threshold of *P* = 0.05 was used to select variables in the final models. Because we tested 2 primary hypotheses (for reflux symptoms with and without erosive esophagitis as outcome variables), we corrected a statistical significance level to *P* = 0.025 (= 0.05/2) by simple Bonferroni correction.

BMI, body mass index; CI, confidence interval; OR, odds ratio;

Supplemental Table 3. Relationship of body mass index with the presence of reflux symptoms without FD symptoms or the presence of FD symptoms without reflux symptoms.

|  |  |  | Presence of reflux symptoms without FD symptoms | | | |
| --- | --- | --- | --- | --- | --- | --- |
|  | No. of | No. of | Univariable analysis  OR (95% CI) | *P* value | Multivariable analysis*  OR (95% CI) | *P* value |
|  | cases | Reflux symptoms |  |  |  |  |
| BMI |  |  |  |  |  |  |
| <18.5 kg/m^2^ | 440 | 6 (1.4%) | 0.44 (0.17-0.91) | *P*=0.03 | 0.64 (0.25-1.35) | *P*=0.26 |
| 18.5≤ to <25 kg/m^2^ | 5032 | 154 (3.0%) | 1 (ref) |  | 1 (ref) |  |
| ≥25 kg/m^2^ | 1640 | 87 (5.3%) | 1.77 (1.35-2.32) | *P*<0.0001 | 1.40 (1.06-1.85) | *P*=0.02 |
|  |  |  | Presence of FD symptoms without reflux symptoms | | | |
|  | No. of | No. of | Univariable analysis  OR (95% CI) | *P* value | Multivariable analysis*  OR (95% CI) | *P* value |
|  | cases | FD symptom |  |  |  |  |
| BMI |  |  |  |  |  |  |
| <18.5 kg/m^2^ | 440 | 35 (8.0%) | 2.43 (1.64-3.50) | *P*<0.0001 | 1.94 (1.29-2.86) | *P*=0.002 |
| 18.5≤ to <25 kg/m^2^ | 5032 | 173 (3.4%) | 1 (ref) |  | 1 (ref) |  |
| ≥25 kg/m^2^ | 1640 | 33 (2.0%) | 0.57 (0.39-0.83) | *P*=0.002 | 0.63 (0.42-0.92) | *P*=0.02 |

*The odds ratio was initially included age group, sex, presence of current smoking, presence of alcohol consumption ≥20g/day, presence of erosive esophagitis, presence of atrophic gastritis, presence of hiatal hernia, and presence of high STAI score. A backward stepwise elimination with a threshold of *P* = 0.05 was used to select variables in the final models. Because we tested 2 primary hypotheses (for reflux symptoms without FD symptom and without FD symptom without reflux symptom as outcome variables), we corrected a statistical significance level to *P* = 0.025 (= 0.05/2) by simple Bonferroni correction.

BMI, body mass index; CI, confidence interval; FD, functional dyspepsia; OR, odds ratio;

Supplemental Table 4. Relationship of body mass index with the presence of reflux symptoms without FD symptoms or the presence of FD symptoms without reflux symptoms stratified by sex.

|  | **Men** | | | **Women** | | |
| --- | --- | --- | --- | --- | --- | --- |
|  | % of cases | Multivariable analysis*  OR (95% CI) | *P* value | % of cases | Multivariable analysis*  OR (95% CI) | *P* value |
| BMI |  | Presence of reflux symptoms without FD symptoms (Outcome variable) | | | | |
| <18.5 kg/m^2^ | 2.3%  (2/89) | 0.76 (0.12-2.46) | *P*=0.69 | 1.1%  (4/351) | 0.60 (0.18-1.53) | *P*=0.31 |
| 18.5≤ to <25 kg/m^2^ | 3.8%  (116/3073) | 1 (ref) |  | 1.9%  (38/1959) | 1 (ref) |  |
| ≥25 kg/m^2^ | 5.7%  (75/1311) | 1.34 (0.98-1.81) | *P*=0.06 | 3.7%  (12/329) | 1.73 (0.84-3.31) | *P*=0.13 |
| BMI |  | Presence of FD symptoms without reflux symptoms (Outcome variable) | | | | |
| <18.5 kg/m^2^ | 6.7%  (6/89) | 2.68 (1.01-5.92) | *P*=0.04 | 8.3%  (29/351) | 1.79 (1.13-2.77) | *P*=0.01 |
| 18.5≤ to <25 kg/m^2^ | 2.8%  (86/3073) | 1 (ref) |  | 4.4%  (87/1959) | 1 (ref) |  |
| ≥25 kg/m^2^ | 1.8%  (24/1311) | 0.62 (0.38-0.97) | *P*=0.04 | 2.7%  (9/329) | 0.67 (0.31-1.28) | *P*=0.24 |

*The odds ratio was initially included age group, sex, presence of current smoking, presence of alcohol consumption ≥20g/day, presence of erosive esophagitis, presence of atrophic gastritis, presence of hiatal hernia, and presence of high STAI score. A backward stepwise elimination with a threshold of *P* = 0.05 was used to select variables in the final models. Because we tested 2 primary hypotheses (for reflux symptoms without FD symptom and without FD symptom without reflux symptom as outcome variables), we corrected a statistical significance level to *P* = 0.025 (= 0.05/2) by simple Bonferroni correction.

BMI, body mass index; CI, confidence interval; FD, functional dyspepsia; OR, odds ratio;
