# Supplementary material for: Influence of sex on the association between body mass index and frequency of upper gastrointestinal symptoms
Source: JGH Open. 2020 Jun 5;4(5):937–44. doi: 10.1002/jgh3.12368 (PMC7578286; doi:10.1002/jgh3.12368)
Supplement: Supplementary file 1 — Table S1. Details of overlap of upper abdominal symptoms stratified by body mass index in the subjects having upper abdominal symptoms of the frequency more than 1 day /week. Table S2. Relationship between body mass index and the frequencies of reflux symptoms more than once a week according to the presence or absence of erosive esophagitis. Table S3. Relationship of body mass index with the presence of reflux symptoms without FD symptoms or the presence of FD symptoms without reflux symptoms. Table S4. Relationship of body mass index with the presence of reflux symptoms without FD symptoms or the presence of FD symptoms without reflux symptoms stratified by sex. [file JGH3-4-937-s001.zip › JGH3_12368_supporting_Information.docx]

**Supporting File 1. Upper gastrointestinal disease study questionnaire**

Questionnaire about abdominal symptoms

1. In the last 3 months, how often did you feel heartburn (an unpleasant stinging or burning sensation in the chest) and/or acid reflux (the sensation of regurgitating small quantities of gastric acid from the stomach up to the throat)?

1□never

2□less than one day a month

3□one day a month

4□two to three days a month

5□one day a week

6□more than one day a week

7□every day

1. In the last 3 months, how often were you unable to finish a regular-sized meal? Also, how often did you feel uncomfortably full after a regular-sized meal?
   1□never

2□less than one day a month

3□one day a month

4□two to three days a month

5□one day a week

6□more than one day a week

7□every day

1. Have you had this inability to finish regular-sized meals and/or this uncomfortable fullness 6 months or longer?
   1□yes
   2□no
2. In the last 3 months, how often did you have pain or burning in the middle of your abdomen above your belly button but not in your chest?
   1□never

2□less than one day a month

3□one day a month

4□two to three days a month

5□one day a week

6□more than one day a week

7□every day

1. Have you had this pain or burning 6 months or longer?
   1□yes
   2□no
2. Please mark the appropriate response option about your examination history of *Helicobacter pylori* infection.
   1□I have not examined *Helicobacter pylori* infection

2□I was diagnosed as positive (infected) for *Helicobacter pylori* infection

3□I was diagnosed as negative (not infected) for *Helicobacter pylori* infection

4□I am not sure

1. To those who chose #2 (positive for *Helicobacter pylori* infection) in question 6, please mark the appropriate response option about the treatment history of *Helicobacter pylori* infection.
   1□I have not received eradication therapy of *Helicobacter pylori* infection

2□I received eradication therapy of *Helicobacter pylori* infection and eventually succeeded in eradication (negative for *Helicobacter pylori* infection)

3□I received eradication therapy of *Helicobacter pylori* infection but did not succeeded in eradication (positive for *Helicobacter pylori* infection)

4□I am not sure

Questionnaire about height/weight, smoking, drinking, lifestyle

- 1. Height cm Body Weight kg
  2. Smoking habits
     1□never
     2□ex-smoker
     3□current smoker
  3. Alcohol consumption
     1□none
     2□less than 20 g/day on average
     3□20 to 60 g/day on average
     4□more than 60 g/day on average
     Alcohol 20 g is the amount of alcohol contained in 180 mL of Japanese sake or 630 mL of beer or two wine glasses of wine or 120 mL of shochu or one whiskey double.
  4. Daily Life
     1□sleep shortage
     2□exercise shortage
     3□irregular meal time
     4□experiencing high level of stress
     5□feeling depressed
  5. This is a question about spinal deformity associated with osteoporosis.
     Does the back of your head touch the wall when you stand with your heels touching the wall?
     1□yes, easily
     2□yes, but not easily
     3□no


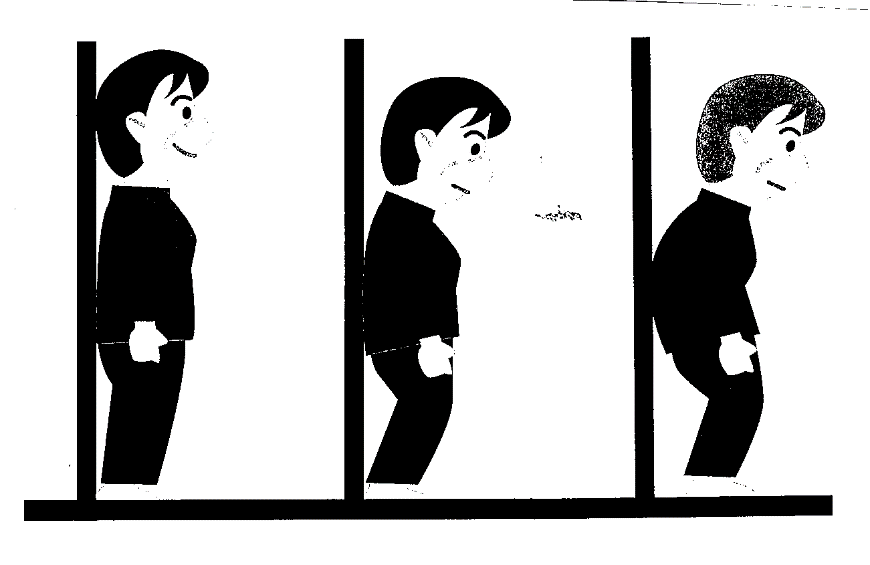


Frequency Scale for the Symptoms of GERD Questionnaire
Do you have any of the following symptoms?
If so, please circle the appropriate number to indicate how you feel.

| Question | | Frequency | | | | |
| --- | --- | --- | --- | --- | --- | --- |
|  |  | Never | Occasionally | Sometimes | Often | Always |
| 1 | Do you get heartburn? | 0 | 1 | 2 | 3 | 4 |
| 2 | Does your stomach get bloated? | 0 | 1 | 2 | 3 | 4 |
| 3 | Does your stomach ever feel heavy after meals? | 0 | 1 | 2 | 3 | 4 |
| 4 | Do you sometimes rub your chest with your hand? | 0 | 1 | 2 | 3 | 4 |
| 5 | Do you ever feel sick after meal? | 0 | 1 | 2 | 3 | 4 |
| 6 | Do you get heartburn after meals? | 0 | 1 | 2 | 3 | 4 |
| 7 | Do you have an unusual (e.g. burning) sensation in your throat? | 0 | 1 | 2 | 3 | 4 |
| 8 | Do you feel full while eating meals? | 0 | 1 | 2 | 3 | 4 |
| 9 | Do some things get stuck when you swallow? | 0 | 1 | 2 | 3 | 4 |
| 10 | Do you get bitter liquid (acid) coming up into your throat? | 0 | 1 | 2 | 3 | 4 |
| 11 | Do you burp a lot? | 0 | 1 | 2 | 3 | 4 |
| 12 | Do you get heartburn if you bend over? | 0 | 1 | 2 | 3 | 4 |

State-Trait Anxiety Inventory Questionnaire
Please read each statement and then circle the appropriate number to the right of the statement to indicate how you generally feel. Do not spend too much time on any one statement but give the answer that seems to describe how you generally feel.

| No | Question | ALMOST NEVER | SOMETIMES | OFTEN | ALMOST ALWAYS |
| --- | --- | --- | --- | --- | --- |
| 1 | I feel pleasant | 1 | 2 | 3 | 4 |
| 2 | I tire quickly | 1 | 2 | 3 | 4 |
| 3 | I feel like crying | 1 | 2 | 3 | 4 |
| 4 | I wish I could be as happy as others seem to be | 1 | 2 | 3 | 4 |
| 5 | I am losing out on things because I can’t make up my mind soon enough | 1 | 2 | 3 | 4 |
| 6 | I feel rested | 1 | 2 | 3 | 4 |
| 7 | I am “calm, cool, and collected” | 1 | 2 | 3 | 4 |
| 8 | I feel that difficulties are piling up so that I cannot overcome them | 1 | 2 | 3 | 4 |
| 9 | I worry too much over something that doesn’t really matter | 1 | 2 | 3 | 4 |
| 10 | I am happy | 1 | 2 | 3 | 4 |
| 11 | I am inclined to take things hard | 1 | 2 | 3 | 4 |
| 12 | I lack self-confidence | 1 | 2 | 3 | 4 |
| 13 | I feel secure | 1 | 2 | 3 | 4 |
| 14 | I try to avoid facing a crisis or difficulty | 1 | 2 | 3 | 4 |
| 15 | I feel blue | 1 | 2 | 3 | 4 |
| 16 | I am content | 1 | 2 | 3 | 4 |
| 17 | Some unimportant thought runs through my mind and bothers me | 1 | 2 | 3 | 4 |
| 18 | I take disappointments so seriously that I can’t put them out of my mind | 1 | 2 | 3 | 4 |
| 19 | I am a steady person | 1 | 2 | 3 | 4 |
| 20 | I become tense and upset when I think about my present concerns | 1 | 2 | 3 | 4 |
